# Phenology of deer ked (*Lipoptena cervi*) host-seeking flight activity and its relationship with prevailing autumn weather

**DOI:** 10.1186/s13071-016-1387-7

**Published:** 2016-02-20

**Authors:** Atle Mysterud, Knut Madslien, Anders Herland, Hildegunn Viljugrein, Bjørnar Ytrehus

**Affiliations:** Department of Biosciences, Centre for Ecological and Evolutionary Synthesis (CEES), University of Oslo, P.O. Box 1066 Blindern, Oslo, NO-0316 Norway; Norwegian Veterinary Institute, P.O. Box 750 Sentrum, Oslo, NO-0106 Norway; Norwegian Institute for Nature Research (NINA), PO Box 5685 Sluppen, Trondheim, NO-7485 Norway

**Keywords:** Deer ked, Invading parasites, Climate, Temperature, Moose, Phenology

## Abstract

**Background:**

The deer ked (*Lipoptena cervi*) is an ectoparasite on cervids that has invaded large parts of Norway, Sweden and Finland during recent decades. During their host-seeking flight activity, the adult deer keds constitute a considerable nuisance to people and limit human outdoor recreation. The bites of the deer ked can cause long-lasting dermatitis in humans. Determining the pattern of flight activity during autumn is hence important.

**Methods:**

Data on flight phenology was gathered by walking along transects in the forest in two counties of Norway during 2009–2013, counting the number of host-seeking keds. We analysed how the flight activity of deer keds varied depending on date and prevailing weather during autumn.

**Results:**

The best model of flight activity included both date and temperature, both as nonlinear terms. Host-seeking deer keds were observed from early August to mid-November with a marked peak in late September. Number of host-seeking keds declined with temperatures falling below the mean, but did not increase much at above mean temperatures. The pattern of flight phenology was similar across the two counties and five years.

**Conclusions:**

Parasitic arthropods may be strongly affected by prevailing weather during off-host periods. Our study shows an estimated positive effect of temperature on deer ked flight activity mainly for below mean temperatures in late autumn, while the effect of temperature on flight activity in early autumn was weak. The pattern of host-seeking flight activity during late, rather than early autumn, is hence more likely to change with ongoing climate change, with a predicted increase in duration of the host-seeking period.

## Background

The deer ked *Lipoptena cervi* (Diptera: Hippoboscidae) is a blood sucking ectoparasite of cervids that has become very common in large areas of Norway, Sweden and Finland in recent years [1, 2]. Deer ked infestation intensity can be high on cervids, especially on moose, the main host in Fennoscandia [3–5]. Modeling suggest that moose density is an important factor for invasion rate [6]. The densities of moose have increased much during the last 50 years in all three countries [7]. In Norway, the number of moose harvested annually have increased from 6–7000 in 1960s to fairly stable around ~35 000 in the last decade [8]. The current spatial variation in moose density within the most affected areas of Norway was not a predictor of deer ked density on host [3], suggesting moose densities in these areas in general are sufficiently high not to limit the deer keds to a large extent. The role of climatic variables is hence likely important.

The deer ked imagines shed their wings when finding a suitable host, preferably a cervid. For the rest of their life, the wingless adults live in the deep layers of the coat of their host, feeding on its blood and reproducing. The deer ked is viviparous, meaning that the offspring develop to mature third-stage larvae within the uterus of the female. The larvae rapidly pupate upon release into the coat of the host, fall passively to the ground and remain there until winged imagines emerge from their pupae during late summer and autumn to search for a new host [9]. After emerging, the imagines seem to use an ambush tactic; they sit in the vegetation, waiting for a host to appear within flight range [10]. When an object with some similarity to a cervid appears, they fly to the presumed host, shed or tear off their wings and burrow their way into the underwool of the coat of the animal and cling to it. Attacking deer keds are a considerable nuisance to domestic and semi-domestic animals [11] and people [12], limiting outdoor recreational activities [13]. They seem to act as vectors for at least one *Bartonella* spp. with a zoonotic potential [14], which is vertically transmitted between the generations of keds [15, 16]. The bites of the deer ked occasionally cause dermatitis in humans [17–19] and dogs [20]. There have been studies on the effect of temperature on off-host survival and pupal development [2, 21–23] and how range expansion may relate to spring and summer temperatures [2], but a study linking host-seeking flight phenology of deer ked in autumn to prevailing weather is lacking.

We analyse here five years of data from two counties of Norway to quantify the extent to which prevailing weather affect host-seeking activity. Based on experimental laboratory evidence [9], we predicted synchrony in flight activity in fall, and that the peak in flight activity across years coincides with a decline in temperature rather than photoperiod (date).

## Methods

### Study area

The study was performed at two different study areas in the southern part of eastern Norway; Nannestad and Eidsvoll municipalities (60°16'N, 11°07'E) in Akershus county and Eidsberg and Marker municipalities (59°35'N, 11°35'E) in Østfold county. The centres of the two study areas are approximately 80 km apart from each other. These study areas were selected because they were presumed to have different deer ked density and history of colonisation [1] and different elevation. The Akershus study site consists of a boreal forest plateau and farmland about 200 m above sea level, surrounded by forest-covered hills ranging up to 400 m above sea level. Relatively rich farmland on marine deposits dominates the area [24]. The forested part is dominated by Scots pine (*Pinus sylvestris*) and Norway spruce (*Picea abies*), and to some extent birch (*Betula pubescent*, *Betula pendula*). In addition, grey alder (*Alnus incana*), aspen (*Populus tremula*), rowan (*Sorbus aucuparia*) and goat willow (*Salix caprea*) are found at lower densities in all parts of the forested area. The deer ked population was established, but at low density, and the area was close to the northwestern border of deer ked distribution range at the time of study. The Østfold study site consists of boreal forest and a high proportion of farmland about 140–170 m above sea level. The forested parts of the two study areas resemble each other. The deer ked population is well established and of high density, and has been present since the late 1980s [1].

### Data collection

The research was conducted from end of July to mid-November 2009–2010 in Akershus county and 2009–2013 in Østfold county, Norway. The phenology of deer ked flight activity was measured as the number of winged deer keds found on the fleece jacket of a human walking slowly in the forest along a predetermined transect formed as a triangle. A joint field trip was done to ensure as similar walking speed as possible among field workers. The triangles had sides of approximately 350 m, i.e. a total length of ~1 km each. In Akershus, six triangles were established on the lowland plateau and two on the surrounding hills. Four triangles were randomly chosen each day monitoring was performed. Due to logistics, the triangles in the hills were always surveyed first or last. In Østfold, four different triangles were established and all of them were surveyed every time. The study person was wearing a black fleece jacket (Stormberg, Kristiansand, Norway, XL-size) and navigated through the triangle by following a prefixed route with a handheld GPS-device (Garmin, GPSmap 60 CSx). With 50 m intervals, the study person halted and carefully took of the jacket. Deer keds sitting on the surface of the jacket and those escaping by flying away were counted. The surveys were conducted during daylight, typically between 8 a.m. and 4 p.m. During the experimental day, study persons avoided use of cosmetics and other chemicals.

### Weather data

Data on daily mean temperature and precipitation was extracted from a grid covering the entire study area (http://www.senorge.no) with spatial resolution of 1 × 1 km and available from the Norwegian Meteorological Institute (ftp://ftp.met.no/projects/klimagrid/tam/). Temperature (mean) and precipitation are estimated for each grid cell based on a net of weather stations recording temperature and precipitation.

### Statistical analysis

We used a negative binomial model in the glmmADMB package [25], as is common to use for parasite data to ensure an appropriate model fit. We used a zero-inflated model with “transect” as a random term to account for more zeroes than expected from a negative binomial model and repeated sampling in the same transects, respectively. We tested for non-linearities using GAM-plotting (library mgcv) in order to choose appropriate parameterizations as a starting point for model selection. Candidate covariates were Julian date (continuous), county (Akershus/Østfold), year (categorical), and mean daily temperature and precipitation. We selected the model with the lowest Akaike Information Criterion (AIC) and at the same time with lowest degrees of freedom (df) within ΔAIC = 2 as recommended [26]. All analyses were done in R version 3.1.3 [27].

## Results

**Fig. 1:**
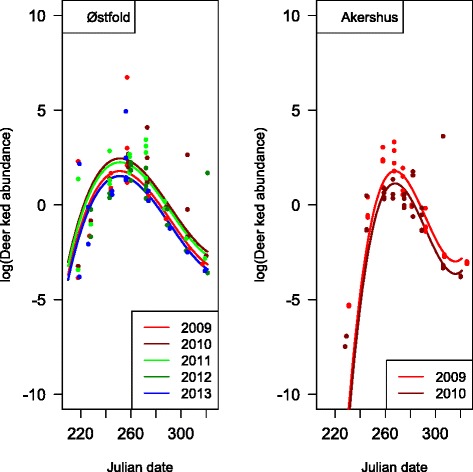
Phenology of deer ked swarming during years 2009–2013 in Østfold and Akershus counties, Norway

**Fig. 2:**
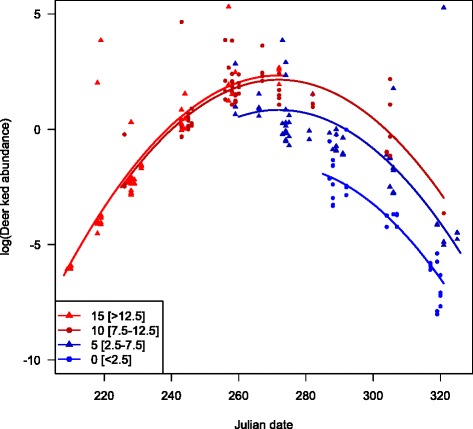
Phenology of deer ked swarming during autumn as a function of temperature. Lines are model predictions for 0, 5, 10 and 15 °C, and only plotted for the dates for which such temperatures were observed. The numbers in brackets are temperature range of observed values for the plotted residuals

The deer ked flight activity season lasted from early August (earliest recorded 6th of August) to mid-November (latest recorded 16th of November) with a peak in last half of September. The model selection retained a second order term for both Julian date and mean daily temperature (Table 1). Note that there was a high negative correlation (r = −0.863) between Julian date and mean daily temperature, and hence estimates of temperature effects are residual effects not captured by date. The flight phenology showed a marked peak that was consistent among years and regions (Fig. 1). There was an effect of temperature that was stronger below than above mean temperature (Fig. 2), and hence mainly an effect of temperature late in the season when temperature was lower and more variable. Neither region nor year as categorical entered the best model (Table 1). The model adding mean daily precipitation was competitive judged from AIC, but with one more df. If added to the model, effect of precipitation was negative in direction, but not significant.

**Table 1:** Parameter estimates of the best negative binomial model explaining the flight phenology of deer keds in boreal forests in Akershus and Østfold counties, Norway, 2009–2013. Under the “adding” and “removing” parameters is reported degrees of freedom (df) and difference in AIC (ΔAIC) relative to the best model (AIC = 634.4, df = 8). Jdate = Julian date (continuous), Temp = mean daily temperature (°C), Prec = mean daily precipitation (mm)

Parameter	Estimate	Std. Error	Z	*P*	df	ΔAIC
Intercept	−159.000	19.600	−8.07	<0.001		
Jdate	1.150	0.145	7.97	<0.001		
(jdate)^2^	−0.002	0.000	−7.97	<0.001		
Temp	0.593	0.144	4.14	<0.001		
(temp)^2^	−0.022	0.009	−2.59	0.010		
Adding						
Prec					9	−0.14
County					9	0.76
Year(cat)					12	5.33
Removing						
(temp)^2^					7	5.58
temp + (temp)^2^					6	28.07
(jdate)^2^					7	67.20
jdate + (jdate)^2^					6	65.51

## Discussion

Climate and environmental temperatures are among the most important limiting factors for the life history and ecology of ectotherm arthropods, impacting physiology, phenology, abundance and in turn distribution. Ectoparasitic arthropods are strongly affected by direct effects of temperature and humidity during off-host periods, while they to a much larger extent are buffered against environment extremes during host infestation periods [28]. The wingless imagines of the deer ked live sheltered on the host, but the pupae on the ground are exposed to harsh climate. When the new generation of imagines emerges from the pupae in the late summer and autumn, they are found as winged adults for whom it is critical to find a host before their energy reserves is depleted. These two off-host periods, pupal stage on ground and the host-seeking flight period of the imagines, are the main critical stages of their life-cycle in terms of exposure to prevailing weather. High cold tolerance through the entire year was found for the deer ked pupae under controlled laboratory conditions [21, 22], but a high temperature peak during pupal winter diapause lowered encapsulation capacity of emerged adults [29]. Determining the pattern of host-seeking flight activity during autumn is also an aspect with relevance for expansion of the deer ked population, in particular since seasonal length are expected to be extended during global warming.

While there are many studies in general on phenology during spring, the autumn is considered the neglected season in climate effect studies [30]. We found strong effects of temperature of flight phenology of deer ked during autumn. The estimated positive effect of temperature was for host-seeking deer keds mainly for below mean temperatures in autumn (Fig. 2). The weak effect of temperature between 10–20 °C in early autumn on the number of host-seeking deer keds is also interesting and demonstrates the strong effect of date beyond the effect of temperature. The deer ked is evolved to emerge and seek for hosts during autumn. An interesting observation is that this behaviour is very synchronous in autumn despite the highly asynchronous production of pupae from late autumn to early summer [9]. As predicted, high synchrony in the peak flight activity of deer keds was observed also in Norway (Fig. 1). Photoperiod had no effect on emergence under two contrasting daylight regimes in the laboratory, while adults emerged after chilling treatments [9]. In our field study, the strong effect of date beyond the effect of temperature might nevertheless indicate a certain role of photoperiod. The phenology of flight activity of a related species, *Lipoptena mazamae*, in south Carolina revealed a very long duration of the flight activity, from May to December [31]. The stronger seasonality in Fennoscandia may lead to a shorter period of flight activity and a more similar pattern across years (Fig. 1).

## Conclusions

Future development of the deer ked population will depend on several factors. Range expansion of deer keds depends on host, mainly moose, density [6], on spring and summer temperature operating on pupae survival [2], and our study shows that warm weather facilitates host acquisition by winged keds long into late autumn. Since small variations in moose density at current high densities seems unrelated to parasitic burden [3], the development may largely be determined by future temperature changes, for which all predictions point to warmer conditions and hence a longer flight season in autumn [32]. The deer ked flight activity as a nuisance to domestic and semi-domestic animals and people is hence likely to continue and possibly extend further into autumn in the future.
